# Malaria and COVID-19: Common and Different Findings

**DOI:** 10.3390/tropicalmed5030141

**Published:** 2020-09-06

**Authors:** Francesco Di Gennaro, Claudia Marotta, Pietro Locantore, Damiano Pizzol, Giovanni Putoto

**Affiliations:** 1 Operational Research Unit, Doctors with Africa CUAMM, 35121 Padova, Italy; cicciodigennaro@yahoo.it (F.D.G.); g.putoto@cuamm.org (G.P.); 2 Institute of Endocrinology, Università Cattolica del Sacro Cuore, 00168 Rome, Italy; pietro.locantore@icloud.com; 3 Italian Agency for Development Cooperation, Khartoum 79371, Sudan; damianopizzol8@gmail.com

**Keywords:** malaria, SARS-CoV-2, COVID-19, preparedness, Africa, emergency, pandemic

## Abstract

Malaria and COVID-19 may have similar aspects and seem to have a strong potential for mutual influence. They have already caused millions of deaths, and the regions where malaria is endemic are at risk of further suffering from the consequences of COVID-19 due to mutual side effects, such as less access to treatment for patients with malaria due to the fear of access to healthcare centers leading to diagnostic delays and worse outcomes. Moreover, the similar and generic symptoms make it harder to achieve an immediate diagnosis. Healthcare systems and professionals will face a great challenge in the case of a COVID-19 and malaria syndemic. Here, we present an overview of common and different findings for both diseases with possible mutual influences of one on the other, especially in countries with limited resources.

## 1. Background

On 11 March 2020, the WHO declared the outbreak of SARS-CoV-2 to be a pandemic infection. Just a few months earlier, pneumonia from a "new virus" was recorded in China; this ailment would later be identified as coronavirus disease 2019 (COVID-19) [1], a highly lethal viral disease with symptoms ranging from interstitial pneumonia to severe acute respiratory syndrome [2,3]. From February to July, around 12 million people were affected, with 500 thousand deaths occurring worldwide. While the figures related to COVID-19 seem to be slowly decreasing in Europe, with a seeming greater number of paucisymptomatic cases [4]; the epidemic is moving with greater force and aggression in Latin America (namely Mexico and Brazil), the United States of America, Asia (India), and Africa, where in the coming months the rainy season is expected along with the seasonal malaria epidemic.

Malaria—a parasitic disease caused by protozoan parasites of the genus *Plasmodium*, transmitted by mosquitoes of the genus *Anopheles*—is among the top ten causes of death in low-income countries and represents one of the great global health challenges. Although 100 countries worldwide have achieved disease elimination and are now malaria-free, around 300 million malaria cases and 500 thousand deaths still occurred worldwide in 2018, with sub-Saharan Africa bearing the greatest burden [5,6]. 

The association between COVID-19 and malaria epidemics can be devastating, especially in low- and middle-income countries (LMICs). Such countries are characterized by healthcare systems that are already fragile due to weak infrastructures, a scarcity of health workers, and limited financial resources. In this perspective, to avoid indirect short- and long-term effects of the COVID-19 pandemic [7] on malaria control programs and on healthcare systems of countries where the two diseases can coexist, preparedness is critical. For these reasons, in order to explore the current landscape and future outlook for a joint scenario of COVID-19 and malaria and the consequences thereof, here we provide an overview for physicians and public health authorities involved in the front line.

## 2. Epidemiology

### 2.1. Data

Quantifying the real number of SARS-CoV-2 cases is not easy since many different challenges are affecting surveillance systems over the world, from laboratory capacities to delay in case notification [8]. Although the mortality estimate is around 2%, different approaches to death cause monitoring and notification among different countries could affect this estimation [9]. What is for sure is that since the first case of SARS-CoV-2 infection identified in December 2019 in Wuhan—a commercial and university hub that is one of the ten most populous Chinese cities—the epidemic has spread all over the world very rapidly. On 13 January, the first confirmed case of COVID-19 outside China occurred in Thailand [10]. In a few weeks, the coronavirus epidemic had spread to 67 countries, from Italy to Iran, and a global pandemic was declared by the WHO on 11 March 2020 [11,12]. As of 17 August 2020, based on WHO reports, 21,689,832 confirmed cases and 770,273 deaths have been reported globally and are regionally distributed as follows: 6,540,222 cases and 420,753 deaths in the Americas, 1,119,579 cases and 25,633 deaths in Africa, 3,239,237 cases and 204,545 deaths in Europe, 5,606,2010 cases and 118,906 deaths in Asia, and 25,742 cases and 429 deaths in Oceania [4].

Meanwhile, malaria is still considered a huge killer, representing one of the biggest health challenges in the world, especially in contexts of poverty. According to the latest World Malaria Report, there were more than 200 million cases of malaria and almost 500 thousand deaths from malaria globally in 2018; although these figures represent a decrease from 2010, malaria is still one of the main causes of deaths in low-income countries [5]. The African region holds the sad record of having more than 90% of global malaria cases, followed by Southeast Asia and then the Eastern Mediterranean Region. Nineteen countries in sub-Saharan Africa and India share almost 85% of the global malaria burden. Six countries accounted for more than half of all malaria cases worldwide: Nigeria (25%); the Democratic Republic of the Congo (12%); Uganda (5%); and Cote d’Ivoire, Mozambique, and Niger (4% each) [5]. 

What emerged the global level is that at all countries are at a very high risk of COVID-19, while half of the world is at risk of malaria, with a greater risk for sub-Saharan Africa and Southeast Asia [5,13]. Although the extent of the COVID-19 epidemic is still relatively low in sub-Saharan Africa, an explosion of COVID-19 cases in Africa would be devastating given the severe impact this would have on healthcare systems that are already very weak [14]. 

Keeping in mind the reliability of data, the numbers of confirmed COVID-19 cases and deaths in the six countries with the biggest global burden of malaria, as of 17 August 2020, are as follows: 49,068 cases and 975 deaths in Nigeria, 9675 cases and 240 deaths in the Democratic Republic of the Congo, 1500 cases and 13 deaths in Uganda, 17,026 cases and 110 deaths in Cote d’Ivoire, 2855 cases and 19 deaths in Mozambique, and 1167 cases and 69 deaths in Niger [4].

To date, only a few clinical cases of malaria and COVID-19 co-infection [15,16] have been reported in the scientific literature, and wider studies are needed to improve knowledge on this topic.

### 2.2. Transmission and Prevention

The main route of transmission of SARS-CoV-2 is though respiratory droplets, with person-to-person contact by asymptomatic carriers also playing an important role. Accordingly, interhuman transmission can be prevented by (1) using face masks; (2) covering coughs and sneezes with tissues; (3) washing hands regularly with soap or disinfecting with sanitizers containing at least 60% alcohol; (4) avoiding contact with infected people; (5) maintaining a physical distance between people (1.5 m), and (6) refraining from touching eyes, nose, and mouth with unwashed hands [17].

The malaria parasite is mainly transmitted by female *Anopheles* mosquito bite, mainly between dusk and dawn. That is why prevention strategies are currently based on two complementary methods: chemoprophylaxis and protection against mosquito bites. 

## 3. Clinical Manifestation and Diagnosis

The clinical diagnosis for both diseases is based on the patient’s symptoms and findings upon physical examination (Table 1).

Early symptoms of SARS-CoV-2 infection such as myalgia, fever, and fatigue could be confused with symptoms of malaria, leading to problems in early clinical diagnosis, especially where malaria is endemic. Different is the case of severe malaria (caused mainly by *Plasmodium falciparum),* where the predominance of neurological signs and symptoms such as confusion, coma, neurological focal signs, severe anemia, and respiratory difficulties can make us more inclined towards the diagnosis of malaria. Blood chemistry tests can also be confusing, as shown in Table 1; therefore, a laboratory test for both malaria and COVID-19 appears essential [18]. The gold standard for malaria diagnosis is the microscopic examination, which can detect the presence of the *Plasmodium*. However, it is an operator-dependent examination, and the sensitivity and specificity depend on the quality of the reagents, the microscope, and the experience of the technician. A useful alternative is provided by the rapid diagnostic tests (RDTs), which appear particularly useful when a reliable microscopic diagnosis is not available and also have the advantage of being a point-of-care method. Another method for diagnosing malaria is to search for parasite nucleic acids using polymerase chain reaction (PCR). Although this technique may be much more sensitive than smear microscopy, PCR results are often not available quickly enough and are therefore rarely used. However, PCR is useful for confirming the malarial parasite species after the diagnosis has been established by smear microscopy or rapid diagnostic tests (RDTs) [19,20].

On the other hand, for patients with suspected SARS-CoV-2 infection, in addition to clinical and/or radiological signs, the use of RT-PCR to detect SARS-CoV-2 nucleic acid in sputum or throat swabs and lower respiratory tract secretions appears essential for diagnosis [21]. Blood chemistry tests in patients with SARS-CoV-2 infection may show leukocytosis with leukopenia, increased liver function indices AST and ALT, elevated LDH, and especially high D-dimers that may indicate a concomitant pulmonary embolism [22]. Indeed, high levels of D-dimer and more severe lymphopenia have been associated with mortality. Chest computed tomography (CT) scans in COVID-19 patients show opacity of frosted glass with or without consolidating abnormalities, consistent with viral pneumonia. Furthermore, these lung lesions often appear to be bilateral and peripheral, and they involve the lower lobes more frequently. Less common findings include pleural thickening, pleural effusion, and lymphadenopathy [23,24]. Other authors presented the scientific community with controversial cases that had negative RT-PCR tests on oropharyngeal swabs despite the CT results being indicative of viral pneumonia and only subsequent positive nasopharyngeal swabs. This underlines how often there can be a divergence between the clinical and virological findings. In addition, IgA, IgM, and IgG serology tests are available to identify patients with recent or previous infection [25]. Malaria and COVID-19 share symptoms and geographic areas of disease spread. The use of laboratory investigations, and therefore the availability of adequate diagnostic capacity, as well as careful clinical surveillance and management of cases appear crucial in differential diagnosis. Furthermore, *Plasmodium* spp. and SARS-CoV-2 are similar in terms of incubation time. For SARS-CoV-2, an incubation period of 11.5 days has been calculated; for *Plasmodium* spp., the incubation period varies from 7 to 30 days in most cases, with a shorter period observed more frequently for *P. falciparum* and a longer one observed for *P. malaria* [5].

Medical and scientific attention should be paid to the potential of COVID-19/malaria co-infections. It would be recommended, especially in areas where malaria is endemic areas, and also because of the availability of malaria tests [26], to double-screen patients with suggestive symptoms for both COVID-19 and malaria. This could bring benefits in contrasting these two infectious diseases, reducing the death toll, and improving the outcome [5,6,7,8,9,10,11,12,13,14,15,16,17,18,19,20,21,22,23,24,25].

## 4. The Role of ACE 2 Receptor in *Plasmodium* spp. and SARS-CoV-2 Infection 

Another interesting aspect of the correlation between SARS-CoV-2 and *Plasmodium* spp. can be seen by considering the angiotensin-converting enzyme receptor 2 (ACE2), since a genetic deletion or insertion polymorphism leads to a reduced expression of ACE2 by altering the concentration of ACE I and D alleles in both infections. This D/I polymorphism shows an important geographical variation [27] and would explain, although this is yet to be demonstrated, the variable prevalence of COVID-19 infections among the global distribution. In fact, SARS-CoV-2 uses the ACE2 receptor as a means of cell entry and requires the spike protein to be primed by cellular serine protease TMPRSS2 [28]. SARS-CoV-2 spike proteins bind to ACE2 receptors of numerous target cells, particularly the type II alveolar cells [29]. 

In SARS-CoV-2 patients, virus infection likely induces proinflammatory events through the activation of transcription nuclear factor (NF)-kB intracellular signaling pathway, leading to overexpression of cytokines and chemokines. In particular, an increased serum expression of interferon (IFN)-γ, interleukin (IL)-1β, IL-6, IL-12, IL-8, MCP-1, and IFN-γ inducible protein-10 has been demonstrated [30]. Similarly, in SARS-CoV-2 infection, increased serum concentrations of the proinflammatory molecules IL-1β, IFN-γ, IP-10, MCP-1, IL-4, and IL-10 has been demonstrated, with the higher levels being associated with more severe disease [31]. During severe infection, endothelial cell dysfunction induces hypercoagulability due to excessive thrombin generation and reduced fibrinolysis [32]. Moreover, virus-driven downregulation of ACE2 receptors may enhance the effects of angiotensin II, leading to increased thrombogenesis [33]. Some authors, in agreement with the role of ACE in lung infections caused by SARS-CoV-2 [4], have suggested how the ACE D/I genotype may influence the clinical course of the infection. Therefore, the ACE D/I polymorphism can play a role in the spread of COVID-19 and in the outcome of the infection, especially in European populations.

Similarly, for malaria, authors suggest a possible protective role in cerebral malaria due to the "D" allele of the ACE I/D polymorphism under both homozygous and heterozygous conditions [33]. In fact, studies reported how several ACE1 and ACE2 polymorphisms reduce erythrocyte invasion by *P. falciparum* [34], resulting in a "protective" effect influencing the development of the parasite and/or the host’s susceptibility to the disease; this was also reported in children [35,36]. This aspect gains importance considering the great number of malaria deaths due to cerebral damage caused by *Plasmodium falciparum* [37] in sub-Saharan Africa. 

In this scenario, more studies on the role of the D allele and other polymorphisms could help to better understand protective factors and develop intervention strategies. Matching data on prevalence of cerebral malaria, COVID-19, and this polymorphism could also be very interesting.

## 5. Relationship of SARS-CoV-2 Infection and *Plasmodium* spp. with Age

In LMICs, the average life expectancy at birth is low, and a huge burden of morbidity is still due to infectious diseases such as human immunodeficiency virus (HIV), malnutrition, tuberculosis, and malaria, with the coexistence of these conditions being a predictive risk factor of death [38,39]. 

For these reasons, even if it is currently believed that SARS-CoV-2 infection is less aggressive in children, some factors should be taken into account when considering this epidemic in a country with a high incidence of malaria. First of all, as for malaria, children, especially those under 5 years of age, are unfortunately the most affected age-group. Furthermore, economic and behavioral changes due to the COVID-19 pandemic could impact families’ response to malaria and increase malaria morbidity and mortality in children. In fact, the cases of the young and elderly can be closely related. As adults and elderly are more susceptible to COVID-19, the fear of the contagion can cause them to be reluctant to visit healthcare facilities, even when they should bring their children with malaria-like symptoms to healthcare facilities. This could lead to a diagnostic delay and therefore a worse outcome. Especially in rural communities, which may have reduced attention, this will affect children with malaria, making an already vulnerable population even more vulnerable. In general, there could be a reduction in access to healthcare facilities due to the fear of contracting COVID-19 [40]. This could have a negative impact on pediatric care. As adults are increasingly implored adults to stay at home, they may hesitate to take their children to healthcare centers when needed, leading to diagnostic delay and the occurrence of more serious cases [19].

## 6. Antimalarials for Treatment of COVID-19

Although the therapeutic role of chloroquine (CQ) and hydroxychloroquine (HCQ) in COVID-19 infection is still not clear, several authors have underlined the antiviral and anti-inflammatory role of these molecules [41], since their action interferes with the ACE2 cell receptor and has activity against many proinflammatory cytokines (e.g., IL-1 and IL-6) [42]. The association of CQ or HCQ with an antibiotic such as azithromycin has been tested experimentally in the field with controversial results, but clinical trials are still ongoing [43,44,45,46] for an evidence-based utilization. However, it does not appear to have any role as a prophylactic in post-exposure prevention [47].

In the past, HCQ played a big role the treatment of malaria, even if the precise mechanism by which it exhibits activity against *Plasmodium* is still unknown. One hypothesis is that HCQ, like CQ, is a weak base and can exert its effect by concentrating on the acid vesicles of the parasite and inhibiting heme polymerization. However, the use of chloroquine in malaria has been suspended in most endemic areas due to resistance.

## 7. Possible Scenarios for COVID-19 in Countries with High Incidence of Malaria

There is great global concern surrounding the possible scenarios of the effects of the COVID-19 pandemic in Africa. It is prudent to assume that for a long time there will be a big gap between official data and the real situation of the COVID-19 pandemic. In part, the data on COVID-19 cases around the world are markedly unrepresentative due to the global scarcity of testing reagents, limited laboratory capabilities, and poor access to healthcare centers [4,48]. Whatever the scenario will be, what is known is that in emergencies, especially in epidemics, one of the most frequent risks is to neglect, suspend, postpone, or close essential prevention and treatment health services. In the end, the burden of avoidable morbidity and mortality from common pathologies causes more damage and creates more victims of the same epidemic; this particularly impacts mothers and children, making the already fragile part of the population even more vulnerable [48,49,50].

Moreover, in sub-Saharan Africa, there are no national healthcare systems capable of withstanding such a wave of patients suffering from acute respiratory failure as COVID-19 has caused in high-income countries. The number of intensive care beds is limited. To this end, the role of intermediate critical care facilities using frugal technology, already tested to be low-cost and effective in contexts with limited resources on other issues, could also be crucial with COVID-19 [51]. 

The Ebola lesson should be valued for the impact it had on malaria control measures, where a significant reduction in malaria diagnoses (but not deaths from malaria) was observed due to the perceived risk of Ebola contagion resulting in a lower number of people accessing healthcare centers [40,52]. In addition, during the Ebola outbreak, it was estimated that malaria cases in Guinea, Liberia, and Sierra Leone could increase to 1 million in 2014 following the disruption in the distribution of insecticide-treated bed nets (ITNs) [53]. The factors that contributed to that situation, i.e., the similarity of the first symptoms between the two diseases and the fear of contracting them in healthcare facilities, are very similar to what we could expect with COVID-19. 

The impact of the epidemic on the health financing system as a whole should also be considered. A polarization of economic resources happened with Ebola, and this is also being observed with COVID-19. Therefore, in the next month, it could be possible to observe an important decrease in economic and human resources for malaria control programs, with a real risk of reducing prevention. This is turn may result in an increase in the numbers of cases, with a consequent increase in morbidity and mortality. The characteristics of COVID-19 and the previous experiences of the Ebola epidemic indicate the need for malaria-endemic countries to consider measures for preparation and prevention, focusing on not only the threat of COVID-19 but also the possible impact of other diseases, especially malaria [16].

In order to face these possible scenarios, COVID-19 preparedness and response in malaria-endemic countries should be focused on the following:

1. Local staff management, including protection, training, supervision, incentives, and rest shifts, should focus on all factors that together can help to ease the fear of contagion, diminish the anger over the death of colleagues, and contain strikes and protests;
2. Infection prevention and control measures should be applied for healthcare workers at the hospital and peripheral levels, making sure to institute appropriate low-technology measures such as washing hands with sodium hypochlorite, segregation of hospital waste, and proper application of the personal protective equipment;
3. Community engagement is crucial, as an effective communication campaign involving local leaders, indigenous associations, and media can compel the community to conform to the new behaviors (distancing, hand washing, stopping of traditional funeral rites, collaborating in contact tracing, etc.) and therefore in the end be able to retain trust in healthcare structures and operators;
4. Data management and operational research should not be neglected. It is of fundamental importance to monitor trends of routine health services use, maternal child health, TB, HIV, etc. Operational research, especially when carried out with local and international partners, allows healthcare professionals to test ideas [54,55], check intuitions, and answer questions from different perspectives (e.g., epidemiology, organization of health services, and policies).

## 8. Conclusions

Malaria and COVID-19 may have similar aspects and seem to have a strong potential for mutual influence. They have already caused millions of deaths, and the regions where malaria is endemic regions are at risk of suffering from the consequences of COVID-19 due to mutual side effects, such as less access to treatment for patients with malaria due to the fear of access to healthcare centers leading to worse outcomes and diagnostic delays. Moreover, the similar and generic symptoms make it harder to achieve an immediate diagnosis. Healthcare systems and professionals will face a great challenge in case of a syndemic [56,57]. The role of young health professionals, well-motivated and trained in primary care, will also be essential [58] in countries with a high burden of malaria. 

In patients with symptoms such as fever, fatigue, and headache, both malaria and COVID-19 tests should always be performed. According to recent WHO recommendations [59], in the case of challenges due to the COVID-19 pandemic (e.g., supply chain disruption for RDTs, health worker absenteeism, shortage of personal protective equipment) a malaria diagnosis should be considered for all fever cases in endemic countries. On the other hand, patients with COVID-19-related symptoms that negative for malaria must undergo isolation to exclude COVID-19 until repetition of the virological sample, thus reducing the potential risk of transmission.

Even though a COVID-19 outbreak may not occur in the malaria-endemic regions, the WHO has called for ministries of health and national malaria control programs to ensure that malaria control efforts are not disrupted while facing the COVID-19 response [59,60]. Preparedness is the key to tackling any public health crisis, and malaria-endemic countries need to be prepared for the challenges COVID-19 could pose. Finally, from a global perspective, it is necessary to increase and join efforts in order to develop an effective vaccine and it make available for everyone, as this would be the most effective preventive measure for both diseases. 

## Figures and Tables

**Table 1:** Summary of the principal characteristics of COVID-19 and malaria.

	COVID–19	Malaria
Classification	Viral disease	Parasitic disease
Infectious agent	SARS-CoV-2	*Plasmodium falciparum, Plasmodium vivax, Plasmodium ovale, Plasmodium malariae, Plasmodium knowlesi*
Main symptoms	Fever, cough	Fever
Severe clinical disease manifestation	Acute respiratory distress syndrome, acute thromboembolic disease, pulmonary embolism	Confusion, coma, neurologic focal signs, severe anemia, acute respiratory distress syndrome, acute renal failure, pulmonary edema, seizure
Diagnostic tests	Molecular tests (RT-PCR) on upper (nasopharyngeal/oropharyngeal swabs, nasal aspirate, nasal wash, or saliva) or lower respiratory tract (sputum or tracheal aspirate or bronchoalveolar lavage (BAL)) sample; Antigen tests on upper or lower respiratory tract sample; Antibody tests [enzyme-linked immunosorbent assays (ELISA), chemiluminescence assays (CLIA), and lateral flow assays (LFA)).	Microscopic diagnosis on blood smear stained with Giemsa; Antigen detection with RDTs using immunochromatographic methods; Molecular diagnosis by PCR; Serology, using either indirect immunofluorescence (IFA) or ELISA; Drug resistance tests with in vitro tests or PCR molecular characterization.
Laboratory findings	Lymphopenia, increased prothrombin time (PT), increased lactate dehydrogenase (LDH), elevated C-reactive protein (CRP), elevated D-dimer, mildly elevated serum amylase, elevated alanine aminotransferase (ALT) and aspartate aminotransferase (AST)	Anemia, hypoglycemia, alterations in kidney function, hyperbilirubinemia, acid–base disturbances
Chest computed tomography (CT) scan	Ground-glass opacities, crazy-paving pattern	Not required for diagnosis
Transmission	Human-to-human; respiratory droplets	Mosquito vector
Age-group most affected by the severe form of the disease	Adult/Elderly	Children/Pregnancy
Defined treatment	No	Yes
Vaccine	Trials ongoing	Trials ongoing
